# Portfolio Model Considering Normal Uncertain Preference Relations of Investors

**DOI:** 10.3390/e27060585

**Published:** 2025-05-30

**Authors:** Yu Zhou, Chun Yan, Xiangrong Wang

**Affiliations:** College of Mathematics and Systems Science, Shandong University of Science and Technology, Qingdao 266590, China; xrwang2025@163.com

**Keywords:** uncertain preference relationship, additive consistency, normal uncertainty distribution, portfolio models

## Abstract

The paper examines the application of uncertainty theory to portfolio decision making, specifically focusing on constructing portfolio models based on uncertain preference relations. Firstly, we establish the theoretical foundation by introducing the theory of uncertainty, which includes uncertain measure and normal uncertain distribution. Then, building upon Markowitz portfolio theory, we propose an uncertain preference relation prioritization model with chance constraints and an additive consistency portfolio model to facilitate rational decision making in a complex and uncertain financial environment. Furthermore, empirical analysis validates our model’s feasibility, demonstrating its advantages in maximizing returns and minimizing risks.

## 1. Introduction

Since Markowitz (1952) [1], the mean-variance model was introduced, and portfolio theory has become central to finance. This model provides a framework for balancing returns and risks in asset allocation. However, practical applications [2] reveal challenges: Michaud (1989) [3] highlighted its sensitivity to input parameters, while Kanaparthi (2024) [4] demonstrated how uncertainty affects its out-of-sample performance. Recent studies have integrated fuzzy and uncertainty theories to improve the model. For example, Gong et al. (2022) [5] proposed a multi-period model using consistent fuzzy numbers, but these models often overlook investor preference complexity. Zadeh’s fuzzy set theory (1965) [6] addresses uncertainty but struggles with multi-objective decision making. Liu and Liu’s (2010) [7] uncertainty theory offers tools for linguistic fuzziness, providing insights into portfolio optimization.

The main challenge of existing models is the insufficient characterization of investor preference uncertainty [8,9,10]. These models typically assume definite or fuzzy preferences, whereas real-world investor preferences are often uncertain, especially in complex market environments. Tversky and Kahneman’s heuristic method (1974) [11] reveals systematic biases in judgment under uncertainty, and their prospect theory (1979) [12] highlights differences in risk preferences when facing gains versus losses. Secondly, the complexity of multi-objective decision making increases the difficulty of model design. In multiobjective portfolio selection, how to balance goals such as mean, variance, skewness, and liquidity remains a challenge. Although the multi-objective portfolio selection model based on the cloud model proposed by Gong et al. (2021) provides flexibility, it still has deficiencies in dealing with complex preference relations [13]. Finally, the consistency problem in group decision making has not been effectively solved. Although the group decision-making method based on additive consistency and sequential consistency proposed by Chen et al. (2014) improves the defects of the Lee method, it still has limitations in dealing with uncertain preference relations [14].

In order to address the above challenges, it is necessary to establish a new portfolio model, particularly one that considers the normal uncertain preference relation of investors. The normal uncertain preference relation reflects investor decision making under uncertainty, especially in complex market environments. It accurately characterizes risk preferences and expected returns, enhancing model practicability and robustness. For instance, Guo et al. (2024) [15] combined fuzzy preference relations with portfolio theory, proposing a model that demonstrates high robustness to distance thresholds and consistency indices. Additionally, new models require efficient consistency measurement methods for multi-objective and group decision making to ensure reliability and accuracy.

Wang and Xu (2015) [16] introduced extended hesitant fuzzy linguistic preference relations for visual consistency interpretation, while Liu et al. (2015) [17] developed a multiplicative consistency test method for hesitant fuzzy preference relations, improving accuracy and reliability. These studies [18,19] provide valuable references for handling complex preference relations, but further research is needed on integrating the normal uncertain preference relation into multiobjective portfolio models.

In existing studies, the portfolio optimization model based on uncertain yield proposed by Li et al. (2022) [20] combines prospect theory with the perspective of expected utility maximization and designs an improved gray wolf optimization algorithm (GWO), verifying its superiority in solving complex non-smooth and non-concave problems. However, these models [21,22,23] still rely on traditional fuzzy theory when dealing with investor preference relations, failing to fully depict the uncertainty of these relations. Furthermore, the portfolio selection model based on Z-number theory and fuzzy neural networks proposed by Ghahtarani (2021) [24] performs better than traditional models under bubble conditions, but its application in multi-objective decision making and group decision making still requires expansion. The portfolio selection model based on the difference between the fundamental value and the market value of assets proposed by Lv et al. (2024) [25] integrates the Z-number theory to handle uncertainty of the data, offering solutions closer to the actual market conditions. Its consistency measurement method in group decision making requires improvement. While existing models have progressed in handling uncertainty and multiobjective decision making, they still struggle to accurately depict investors’ normal uncertain preference relations.

By introducing the normal uncertain preference relation, the new portfolio model effectively reflects investor behavior under uncertainty, particularly in complex markets. Consider multiple objectives such as mean, variance, skewness, and liquidity, while providing flexibility through compromise programming. An improved consistency measurement method addresses individual preference inconsistencies, improving the reliability of the decision. For example, Gong et al. (2020) [26] introduced linear uncertain preference relations (LUPRs) to address global complementarity and consistency challenges. Fuzzy interval preference relations (IFPRs) play a crucial role in improving group decision-making processes. Future research could explore normal uncertain preference relations in financial decisions and improve model accuracy with machine learning. Nozarpour et al. (2023) [27] developed a multi-period stock portfolio model that considers transaction costs and correction time periods, better reflecting real market conditions. Furthermore, Avramov et al. (2022) [28] examined the impact of uncertainty about ESG on asset pricing, offering new research directions. These studies contribute to a more comprehensive portfolio model and provide practical tools for investors.

This paper proposes a new portfolio model with normal uncertain preference relations to more accurately describe investors’ decision-making behavior under uncertainty. Compared with existing models, this model handles uncertain preference relations and multiobjective decision-making problems more effectively, improves group decision-making reliability and accuracy through an enhanced consistency measurement method, and provides a practical tool for investors to make scientific decisions in complex market environments. Future research can explore the application of normal uncertain preference relations in other financial decisions.

The paper is structured as follows. Section 2 introduces uncertainty theory and defines preference relations. Section 3 formulates uncertain preference relations and their additive consistency. Section 4 develops a weight derivation model for the NUPR. Section 5 demonstrates the methodology with an empirical case of China’s SSE 50 index. Section 6 concludes and outlines future research directions.

## 2. Preliminaries

This section introduces the fundamentals of uncertain preference relations (UPRs) and portfolio theory. We define Θ as a finite set of alternatives {ϑ1,ϑ2,…,ϑn}, where each $\vartheta_{i}\left(i\in \left\{1,2,\ldots ,n\right\}\right)$ represents an alternative.

### 2.1. Uncertainty Theory

Uncertainty theory addresses financial and economic challenges by providing tools to handle practical uncertainties. Unlike conventional theories assuming deterministic or stochastic market behavior, uncertainty theory offers new perspectives in portfolio selection, risk management, and insurance strategy formulation. This paper reviews its progress and applications in finance and economics.

#### 2.1.1. Uncertain Measure

The set L is an algebra on Γ if it satisfies: (a) Γ∈L; (b) for any $\Lambda \in L,\Lambda^{c}\in L$; (c) for any countable sequence $\Lambda_{1},\Lambda_{2},\ldots ,\Lambda_{n}$, their union $\cup_{i=1}^{n}\Lambda_{i}\in L$. If condition (c) is replaced with the ability to handle countably infinite sequences, i.e., when $\Lambda_{1},\Lambda_{2},\ldots \in L$, we have $\cup_{i=1}^{\infty }\Lambda_{i}\in L$, then the resulting collection L is called a σ-algebra on Γ. To establish a reasonable framework for trust analysis, Liu [7] proposed the following axioms:

Normal axiom. For the total set Γ,M{Γ}=1.

Duality axiom. For any event $\Lambda ,M\left\{\Lambda \right\}+M\left\{\Lambda^{c}\right\}=1$.

Subadditive axiom. The sequence of events for each numbered $\Lambda_{1},\Lambda_{2},\ldots$, we have

(1) $$M\left\{\overset{\infty }{\underset{i=1}{⋃}}\Lambda_{i}\right\}\leq \sum_{i=1}^{\infty }M\left\{\Lambda_{i}\right\}.$$

Product axiom. Set $\left(\Gamma_{k},L_{k},M_{k}\right)$ space for uncertainty, for k=1,2,…, where the product uncertainty measure *M* is an uncertainty measure that satisfies the following conditions

(2) $$M\left\{\prod_{k=1}^{\infty }\Lambda_{k}\right\}=\overset{\infty }{\underset{k=1}{⋀}}M_{k}\left\{\Lambda_{k}\right\}.$$

Here, $\Lambda_{k}$ from $L_{k}$ in either event, for k=1,2,….

**Definition** **1** ([7])**.** *An uncertain variable is a function ζ from the indeterministic space (Γ,L,M) to the set of real numbers such that for any Borel set of real numbers B,{ζ∈B} is an event. The uncertainty distribution* Φ *of the uncertain variable ζ is defined as*

(3) α=Φ(x)=M{ζ≤x}.

**Theorem** **1** ([7])**.** *Let $\zeta_{1},\zeta_{2},\ldots ,\zeta_{n}$ be independent uncertain variables with respective probability distributions $\Phi_{1},\Phi_{2},\ldots ,\Phi_{n}$. If the function $f\left(\zeta_{1},\zeta_{2},\ldots ,\zeta_{n}\right)$ is strictly increasing with respect to $\zeta_{1},\zeta_{2},\ldots ,\zeta_{m}$ and strictly decreasing with respect to $\zeta_{m+1},\zeta_{m+2},\ldots ,\zeta_{n}$, then*

(4) $$\xi =f\left(\zeta_{1},\zeta_{2},\ldots ,\zeta_{n}\right).$$

*Has an inverse uncertainty distribution*

(5) $$\Psi^{-1}\left(\alpha \right)=f\left(\Phi_{1}^{-1}\left(\alpha \right),\ldots ,\Phi_{m}^{-1}\left(\alpha \right),\Phi_{m+1}^{-1}\left(1-\alpha \right),\ldots ,\Phi_{n}^{-1}\left(1-\alpha \right)\right).$$

#### 2.1.2. Normal Uncertain Distribution and Its Inverse Distribution

**Theorem** **2** ([7])**.** *An uncertain variable ζ is said to be normal if it has a normal uncertainty distribution*

(6) $$\Phi \left(x\right)={\left(1+\exp\left(\frac{\pi (e-x)}{\sqrt{3}\sigma }\right)\right)}^{-1},$$

**Figure 1:**
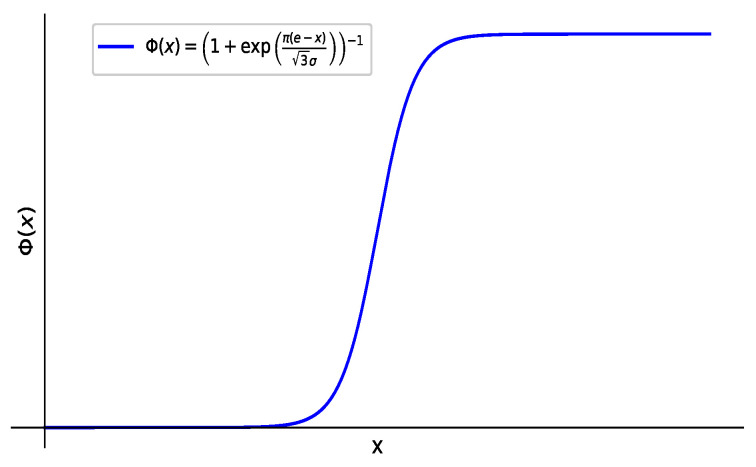
Normal uncertain distribution.

*where N(e,σ) indicates that e and σ are real numbers and σ>0 (see Figure 1).*

**Figure 2:**
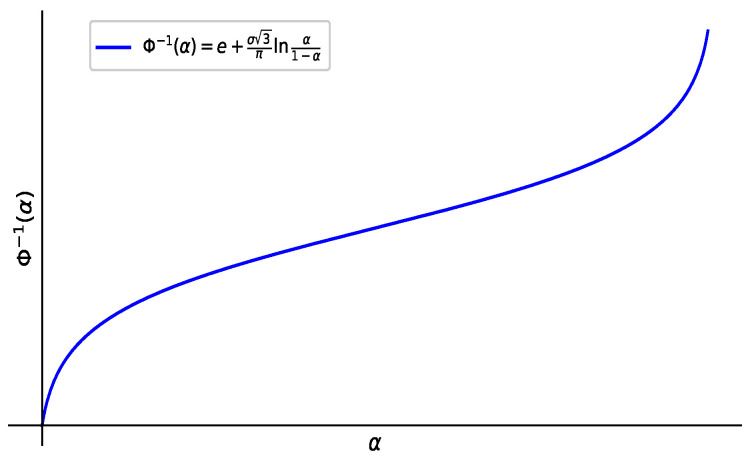
Inverse normal uncertain distribution.

**Theorem** **3** ([7])**.** *The inverse uncertainty distribution for the normal uncertainty variable N(e,σ) is as follows (see Figure 2).*

(7) $$\Phi^{-1}\left(\alpha \right)=e+\frac{\sigma \sqrt{3}}{\pi }\ln\frac{\alpha }{1-\alpha }.$$

### 2.2. Portfolio Theory

We assume that investors are rational, meaning they always aim to optimize the balance between the potential for loss and gain in investment decision-making processes to achieve desired outcomes. Let $R=\left(R_{1},R_{2},\ldots ,R_{n}\right)$ and $e=\left(e_{1},e_{2},\ldots ,e_{n}\right)$ represent the actual and expected returns of *N* securities, respectively. In this context, $R_{i}$ can be regarded as a probabilistic variable with inherent uncertainty. Therefore, $e_{i}$ denotes the expected value of $R_{i}$ denoted as $E\left(R_{i}\right)=e_{i}$, where i∈N. Suppose that the matrix $D\left(R\right)={\left(\sigma_{ij}\right)}_{n\times n}$ represents the stochastic variables $R=\left(R_{1},R_{2},\ldots ,R_{n}\right)$ in the covariance matrix. The weight vector $H=\left\{\eta_{1},\eta_{2},\ldots ,\eta_{n}\right\}$ represents the proportion of investment in *N* securities and satisfies $\sum_{i=1}^{n}\eta_{i}=1$. Refraining from short selling is mandatory when $\eta_{i}\geq 0$ within the security market, as negative values imply involvement in activities related to short selling by participants in the market. For simplicity purposes, this paper only considers scenarios where short selling is not carried out. Taking into account these suppositions, Markowitz [1] formulated the subsequent pair of models.

In the management of the portfolio, if investors have a predetermined expected return, namely $\mu_{0}$, one of its goals by designing a portfolio is to minimize risk. Such a goal can be expressed in the following ways

(8) $$\begin{array}{cc} \min & \sum_{i=1}^{n}\sum_{j=1}^{n}\eta_{i}\sigma_{ij}\eta_{j}\\ s.t. & \left\{\begin{array}{cc} \sum_{i=1}^{n}\eta_{i}e_{i}=e_{0}, & i\in N,\\ \sum_{i=1}^{n}\eta_{i}=1, & i\in N,\\ \eta_{i}\geq 0, & i\in N. \end{array}\right. \end{array}$$

The portfolio strategy assumes that given the known investment risk, namely $\sigma_{0}^{2}$, the investor’s objective is to design a portfolio model that can achieve maximum return at a specific level of risk. This formula can be used to represent the given model.

(9) $$\begin{array}{cc} \max & \sum_{i=1}^{n}\eta_{i}e_{i}\\ s.t. & \left\{\begin{array}{cc} \sum_{i=1}^{n}\sum_{j=1}^{n}\eta_{i}\sigma_{ij}\eta_{j}=\sigma_{0}^{2}, & i,j\in N,\\ \sum_{i=1}^{n}\eta_{i}=1, & i\in N,\\ \eta_{i}\geq 0, & i\in N. \end{array}\right. \end{array}$$

## 3. Priority Model of UPR Obeying Normal Uncertainty Distribution

In the rapidly evolving information age, uncertainty has become the norm in investors’ decision-making processes. While traditional probability theory is effective in handling random phenomena, it falls short when confronted with complex and ambiguous information scenarios. The uncertainty theory proposed by Liu [12] is revolutionizing our understanding of and response to uncertainty due to its exceptional adaptability and broad applicability. By introducing concepts like “uncertainty set” and “uncertainty function”, this theory goes beyond traditional probability theory. It addresses not only randomness but also ambiguity, chaos, and incomplete information. It provides a toolkit for solving complex decision problems, whether data are lacking or uncertain, effectively supporting investors’ judgment and actions.

### 3.1. NUPRs and Their Consistency Definitions

In practical decision making, investors express their judgments with pairwise comparisons of alternative options. However, accurately quantifying their preferences poses challenges as only the approximate confidence distribution of investors’ judgments can be captured. In this particular section, we make the assumption that the time period value judgment $\zeta_{ij}$ between alternative options $\vartheta_{i}$ and $\vartheta_{j}$ follows a normal uncertain distribution, i.e., $\zeta_{ij}∼N\left(e_{ij},\sigma_{ij}\right)$. Similar to fuzzy preference relations [23,24,25,26,27,28], the definition of an NUPR is based on uncertainty theory. Confidence-based uncertainty decision making can effectively capture investors’ confidence in their experiential judgments under uncertainty and is particularly suitable for subjective decision-making environments with knowledge and experience constraints. Confidence-based NUPRs extend uncertain preference relations (UPR) and also enable analysis of whether uncertain variables satisfy definitions of complementarity and consistency.

**Definition** **2.** *Consider a matrix $Z={\left(\zeta_{ij}\right)}_{n\times n}$ consisting of non-negative elements, where each $\zeta_{ij}$ represents an uncertain variable and the distribution $\zeta_{ij}$ follows a Gaussian distribution $N\left(e_{ij},\sigma_{ij}\right)$, where $N\left(e_{ij},\sigma_{ij}\right)$ denotes a Gaussian distribution characterized by the average $e_{ij}$ and a standard deviation of $\sigma_{ij}$. The corresponding uncertainty distribution function is denoted as $\Phi_{ij}$, and the inverse uncertainty distribution function is denoted as $\Phi_{ij}^{-1}$. For any confidence level α,0≤α≤1, we have*

*(a) $\Phi_{ii}^{-1}\left(\alpha \right)=0.5$. (self preference stability)*

*(b) $\Phi_{ij}^{-1}\left(\alpha \right)+\Phi_{ji}^{-1}\left(1-\alpha \right)=1,i,j\in N$. (global complementarity)*

*Among them, the $\Phi_{ij}^{-1}\left(\alpha \right)$ can be made of the distribution of the normal uncertain (INCDF) expressions of the inverse cumulative distribution function $\Phi_{ij}^{-1}\left(\alpha \right)=e_{ij}+\frac{\sigma_{ij}\sqrt{3}}{\pi }\ln\frac{\alpha }{1-\alpha }$.*

*The matrix $Z={\left(\zeta_{ij}\right)}_{n\times n}$ is called the normal uncertain preference relation (NUPR) matrix. If all $\zeta_{ij}$ follow normal distribution of uncertainty, the Z called normal uncertain preference relation matrix.*

*The uncertainty preference relation (UPRs) employs the normal distribution to depict the uncertainty of preferences, capturing both the degree of superiority and inferiority between options as well as their associated uncertainties. In this context, we assume that $\zeta_{13}$ and $\zeta_{31}$ are uncertain variables following a normal distribution, such as $\zeta_{13}∼$$N\left(e_{13},\sigma_{13}\right)$ and $\zeta_{31}∼N\left(e_{31},\sigma_{31}\right)$. For example, if $\zeta_{13}∼N\left(0.45,0.015\right)$, it indicates that the preference degree of option $\zeta_{1}$ over $\zeta_{3}$ is normally distributed with an average preference degree of 0.45 and a standard deviation of 0.015. Similarly, if the preference degree of $\zeta_{31}∼N\left(0.65,0.015\right)$, it implies that the preference degree of option $\zeta_{3}$ over $\zeta_{1}$ follows a normal uncertain distribution with an average preference degree of 0.65 and a standard deviation of 0.015.*

**Definition** **3** (NUPRs’ additive consistency)**.** *If for any $\alpha ,0\leq \alpha \leq 1,\Phi_{ik}^{-1}\left(\alpha \right)+$$\Phi_{kj}^{-1}\left(\alpha \right)=\Phi_{ij}^{-1}\left(\alpha \right)+0.5,i<k<j,i,j,k\in N$. Then, the NUPR $Z={\left(\zeta_{ij}\right)}_{n\times n}$ is said to have additive consistency. Assume that X is an uncertain variable that follows a normal uncertain distribution [12], that is, X∼N(e,σ). Its distribution function for Φ(x)=${\left(1+\exp\left(\frac{\pi (e-x)}{\sqrt{3}\sigma }\right)\right)}^{-1},x\in R,e,\sigma \in R,\sigma >0$. If Φ(x) is a standard normal uncertain distribution function, according to the 3σ principle, the confidence that X falls in the interval [e−3σ,e+3σ] is 99.14%. Then, the uncertain variable X of the normal uncertain distribution can be approximately replaced by the interval $\vartheta =\left[\vartheta^{-},\vartheta^{+}\right]$, and their relationship is as follows*

(10) $$\left[\vartheta^{-},\vartheta^{+}\right]=\left[e-3\sigma ,e+3\sigma \right],e=\frac{\vartheta^{-}+\vartheta^{+}}{2},\sigma =\frac{\vartheta^{+}-\vartheta^{-}}{6}.$$

*For example, according to the principle of 3σ, the interval [0.35,0.65] can be approximately and equivalently transformed into an uncertain variable X that follows the normal uncertain distribution, satisfying X∼N(0.50,0.05), The interval [0.6,0.9] can be approximately equivalent transformed into an uncertain variable X which follows the normal uncertain distribution and satisfies X∼N(0.75,0.05).*

### 3.2. NUPR Prioritization Model with Opportunity Constraints

The discrete characteristic is evident in the preference relation of each investor when comparing alternatives pairwise, whereas the true value introduces a certain degree of uncertainty. Therefore, it becomes imperative to describe investors’ decision values using uncertain variables that conform to specific distributions. The proposed model is constructed based on the chance constrained programming approach in order to achieve an optimal ranking of investment alternatives.

Consider UPR *Z* with a normal uncertain distribution and its corresponding NUPR $\tilde{Z}$. In the normal uncertain distribution matrix $\tilde{Z}$, each possible value of the uncertain variable ${\tilde{\zeta }}_{ij}$ indicates the degree of preference of the investor regarding the alternative $\vartheta_{i}$ over $\vartheta_{j}$. Here, it is still assumed that $H={\left(\eta_{1},\eta_{2},\ldots ,\eta_{n}\right)}^{T}$ weight vector and the optimal decision (objective function) is the minimum of the deviation between the ideal judgment $\frac{1}{2}\left(\eta_{i}-\eta_{j}+1\right)$ and the uncertain variable ${\tilde{\zeta }}_{ij}$. The chance constraint is the possibility of event occurrence, and the deviation between event $\frac{1}{2}\left(\eta_{i}-\eta_{j}+1\right)$ and the uncertain variable ${\tilde{\zeta }}_{ij}$ does not exceed the threshold $\xi_{ij}$ under the trust degree $\alpha_{ij}$ as a constraint. Therefore, the most suitable ranking model utilizing the NUPR with a normally uncertain distribution and chance constraints can be formulated as.

(11) $$\begin{array}{cc} \min & \sum_{i\neq j,i,j\in N}\xi_{ij}\\ s.t. & \left\{\begin{array}{cc} M\left\{\left|\frac{1}{2}\left(\eta_{i}-\eta_{j}+1\right)-{\tilde{\zeta }}_{ij}\right|\leq \xi_{ij}\right\}\geq \alpha_{ij}, & i,j\in N,\\ {\tilde{\zeta }}_{ij}∼N\left(e_{ij},\sigma_{ij}\right), & i,j\in N,\\ \sum_{i=1}^{n}\eta_{i}=1, & i\in N,\\ \eta_{i},\xi_{ij}\geq 0, & i,j\in N. \end{array}\right. \end{array}$$

where $0.5\leq \alpha_{ij}\leq 1$ is the trust degree that satisfies the constraint of the consistency condition. $\left|\frac{1}{2}\left(\eta_{i}-\eta_{j}+1\right)-{\tilde{\zeta }}_{ij}\right|$ is the normal uncertainty distribution of real variable ${\tilde{\zeta }}_{ij}$ and ideal judge $\frac{1}{2}\left(\eta_{i}-\eta_{j}+1\right)$, the deviation between which we assume is less than the threshold deduced $\xi_{ij}$. The objective function aims to minimize the sum of all thresholds $\xi_{ij}$, considering the confidence degree $\alpha_{ij}$.

**Theorem** **4.** *The target value in Equation (11) is an increasing function of trust.*

**Proof.**  The greater the value of $\alpha_{ij}$ in Equation (11), the larger the threshold of $\xi_{ij}$ or less for meeting events $\left|\frac{1}{2}\left(\eta_{i}-\eta_{j}+1\right)-{\tilde{\zeta }}_{ij}\right|$, therefore indicating that the target min $\sum_{i\neq j,i,j\in N}\xi_{ij}$ shows a positive correlation with the likelihood for all pairs i,j∈N. □

Let us reevaluate the limitations in Equation (11). As per the principles of uncertain systems [12], it is essential to consider chance constraints.

(12) $$M\left\{\left|\frac{1}{2}\left(\eta_{i}-\eta_{j}+1\right)-{\tilde{\zeta }}_{ij}\right|\leq \xi_{ij}\right\}\geq \alpha_{ij},$$

is equivalent to

(13) $$M\left\{\frac{1}{2}\left(\eta_{i}-\eta_{j}+1\right)-{\tilde{\zeta }}_{ij}\leq \xi_{ij}\right\}\geq \alpha_{ij},$$

(14) $$M\left\{-\frac{1}{2}\left(\eta_{i}-\eta_{j}+1\right)+{\tilde{\zeta }}_{ij}\leq \xi_{ij}\right\}\geq \alpha_{ij}.$$

This implies that Equation (11) is indistinguishable from Equation (15).

(15) $$\begin{array}{cc} \min & \sum_{i\neq j,i,j\in N}\xi_{ij}\\ \;s.t.\; & \left\{\begin{array}{cc} M\left\{\frac{1}{2}\left(\eta_{i}-\eta_{j}+1\right)-{\tilde{\zeta }}_{ij}\leq \xi_{ij}\right\}\geq \alpha_{ij}, & i,j\in N,\\ \left\{-\frac{1}{2}\left(\eta_{i}-\eta_{j}+1\right)+{\tilde{\zeta }}_{ij}\leq \xi_{ij}\right\}\geq \alpha_{ij}, & i,j\in N,\\ {\tilde{\zeta }}_{ij}∼N\left(e_{ij},\sigma_{ij}\right), & i,j\in N,\\ \sum_{i=1}^{n}\eta_{i}=1, & i\in N,\\ \eta_{i},\xi_{ij}\geq 0, & i,j\in N. \end{array}\right. \end{array}$$

**Theorem** **5.** *A deterministic nonlinear Equation (16) is equivalent to a prioritization Equation (15) with chance constraints, representing a typical scenario of uncertainty.*

(16) $$\begin{array}{cc} \; & \min\sum_{i\neq j,i,j\in N}\xi_{ij}\\ \; & \;s.t.\;\left\{\begin{array}{cc} \Phi_{ij}^{-1}\left(\alpha \right)+\frac{1}{2}\left(\eta_{i}-\eta_{j}+1\right)-2e_{ij}-\xi_{ij}\leq 0, & i,j\in N,\\ 2e_{ij}-\xi_{ij}-\frac{1}{2}\left(\eta_{i}-\eta_{j}+1\right)-\Phi_{ij}^{-1}\left(\alpha \right)\leq 0, & i,j\in N,\\ \sum_{i=1}^{n}\eta_{i}=1, & i\in N,\\ \eta_{i},\xi_{ij}\geq 0, & i,j\in N. \end{array}\right. \end{array}$$

**Proof.**  In Equation (15), for ${\tilde{\zeta }}_{ij}∼N\left(e_{ij},\sigma_{ij}\right)$, the chance constraint satisfies

(17) $$\begin{array}{cc} \;\;Equation(13)\Leftrightarrow \; & M\left\{-{\tilde{\zeta }}_{ij}\leq -\frac{1}{2}\left(\eta_{i}-\eta_{j}+1\right)+\xi_{ij}\right\}\geq \alpha_{ij}, \end{array}$$

(18) $$\begin{array}{cc} \;\;\;\;\;\;\;\;\;\;\;\;\;\;\;\;\;\;\;\;\;\;\;\;\;\;\Leftrightarrow \; & 1-M\left\{{\tilde{\zeta }}_{ij}\leq \frac{1}{2}\left(\eta_{i}-\eta_{j}+1\right)-\xi_{ij}\right\}\geq \alpha_{ij}, \end{array}$$

(19) $$\begin{array}{cc} \;\;\;\;\;\;\;\;\;\;\;\;\;\;\;\;\Leftrightarrow \; & \Phi \left(\frac{1}{2}\left(\eta_{i}-\eta_{j}+1\right)-\xi_{ij}\right)\leq 1-\alpha_{ij}, \end{array}$$

(20) $$\begin{array}{cc} \;\;\;\;\;\;\;\;\;\;\;\;\;\;\;\;\;\;\;\;\;\;\;\;\;\;\;\;\;\;\;\;\;\;\;\;\;\;\;\;\;\;\;\;\;\;\;\;\;\;\;\;\;\;\Leftrightarrow \; & {\left(1+\exp\left(\frac{\pi \left(e_{ij}-\frac{1}{2}\left(\eta_{i}-\eta_{j}+1\right)+\xi_{ij}\right)}{\sqrt{3}\sigma_{ij}}\right)\right)}^{-1}\leq 1-\alpha_{ij}, \end{array}$$

(21) $$\begin{array}{cc} \;\;\;\;\;\;\;\;\;\;\;\;\;\;\;\;\;\;\;\;\;\;\;\;\;\;\;\;\;\;\;\;\;\;\;\;\;\;\;\;\Leftrightarrow \; & \exp\left(\frac{\pi \left(e_{ij}-\frac{1}{2}\left(\eta_{i}-\eta_{j}+1\right)+\xi_{ij}\right)}{\sqrt{3}\sigma_{ij}}\right)\geq \frac{\alpha_{ij}}{1-\alpha_{ij}}, \end{array}$$

(22) $$\begin{array}{cc} \;\;\;\;\;\;\;\;\;\;\;\;\;\;\;\;\;\;\;\;\;\;\;\;\;\;\;\;\;\;\;\;\;\;\;\Leftrightarrow \; & e_{ij}+\xi_{ij}-\frac{1}{2}\left(\eta_{i}-\eta_{j}+1\right)\geq \frac{\sqrt{3}\sigma_{ij}}{\pi }\ln\frac{\alpha_{ij}}{1-\alpha_{ij}}, \end{array}$$

(23) $$\begin{array}{cc} \;\;\;\;\;\;\;\;\;\;\;\;\;\;\;\;\;\;\;\;\;\;\;\;\;\;\;\;\;\;\;\;\;\;\;\;\;\;\;\;\;\;\;\;\;\Leftrightarrow \; & -e_{ij}-\xi_{ij}+\frac{1}{2}\left(\eta_{i}-\eta_{j}+1\right)+\frac{\sqrt{3}\sigma_{ij}}{\pi }\ln\frac{\alpha_{ij}}{1-\alpha_{ij}}\leq 0, \end{array}$$

(24) $$\begin{array}{cc} \;\;\;\;\;\;\;\;\;\;\;\;\;\;\;\;\;\;\;\;\;\;\;\;\;\;\;\;\Leftrightarrow \; & \Phi_{ij}^{-1}\left(\alpha \right)+\frac{1}{2}\left(\eta_{i}-\eta_{j}+1\right)-2e_{ij}-\xi_{ij}\leq 0. \end{array}$$

By the same token,

(25) $$\begin{array}{cc} Equation(14)\Leftrightarrow \; & M\left\{{\tilde{\zeta }}_{ij}\leq \frac{1}{2}\left(\eta_{i}-\eta_{j}+1\right)+\xi_{ij}\right\}\geq \alpha_{ij}, \end{array}$$

(26) $$\begin{array}{cc} \;\;\;\;\;\;\;\;\;\;\;\;\;\;\;\;\;\;\;\;\;\;\;\;\;\;\;\;\;\;\;\;\;\;\;\Leftrightarrow \; & 1-M\left\{-{\tilde{\zeta }}_{ij}\leq -\frac{1}{2}\left(\eta_{i}-\eta_{j}+1\right)-\xi_{ij}\right\}\geq \alpha_{ij}, \end{array}$$

(27) $$\begin{array}{cc} \;\;\;\;\;\;\;\;\;\;\;\;\;\;\;\;\;\;\;\;\;\;\;\;\;\;\;\;\;\;\Leftrightarrow \; & M\left\{{\tilde{\zeta }}_{ij}\geq \frac{1}{2}\left(\eta_{i}-\eta_{j}+1\right)+\xi_{ij}\right\}\leq 1-\alpha_{ij}, \end{array}$$

(28) $$\begin{array}{cc} \;\;\;\;\;\;\;\;\;\;\;\;\;\;\Leftrightarrow \; & \Phi \left(\frac{1}{2}\left(\eta_{i}-\eta_{j}+1\right)+\xi_{ij}\right)\geq \alpha_{ij}, \end{array}$$

(29) $$\begin{array}{cc} \;\;\;\;\;\;\;\;\;\;\;\;\;\;\;\;\;\;\;\;\;\;\;\;\;\;\;\;\;\;\;\;\;\;\;\;\;\;\;\;\;\;\;\;\;\;\;\;\;\;\;\;\;\;\;\;\;\;\;\Leftrightarrow \; & {\left(1+\exp\left(-\frac{\pi \left(-e_{ij}+\frac{1}{2}\left(\eta_{i}-\eta_{j}+1\right)+\xi_{ij}\right)}{\sqrt{3}\sigma_{ij}}\right)\right)}^{-1}\geq \alpha_{ij}, \end{array}$$

(30) $$\begin{array}{cc} \;\;\;\;\;\;\;\;\;\;\;\;\;\;\;\;\;\;\;\;\;\;\;\;\;\;\;\;\;\;\;\;\;\;\;\;\;\;\;\;\;\;\;\;\;\;\;\;\;\;\;\;\;\Leftrightarrow \; & \exp\left(-\frac{\pi \left(-e_{ij}+\frac{1}{2}\left(\eta_{i}-\eta_{j}+1\right)+\xi_{ij}\right)}{\sqrt{3}\sigma_{ij}}\right)\leq \frac{1-\alpha_{ij}}{\alpha_{ij}}, \end{array}$$

(31) $$\begin{array}{cc} \;\;\;\;\;\;\;\;\;\;\;\;\;\;\;\;\;\;\;\;\;\;\;\;\;\;\;\;\;\;\;\;\;\;\;\;\;\;\;\;\;\Leftrightarrow \; & e_{ij}-\xi_{ij}-\frac{1}{2}\left(\eta_{i}-\eta_{j}+1\right)\leq \frac{\sqrt{3}\sigma_{ij}}{\pi }\ln\frac{1-\alpha_{ij}}{\alpha_{ij}}, \end{array}$$

(32) $$\begin{array}{cc} \;\;\;\;\;\;\;\;\;\;\;\;\;\;\;\;\;\;\;\;\;\;\;\;\;\;\;\;\;\;\;\;\;\;\;\Leftrightarrow \; & 2e_{ij}-\xi_{ij}-\frac{1}{2}\left(\eta_{i}-\eta_{j}+1\right)-\Phi_{ij}^{-1}\left(\alpha \right)\leq 0. \end{array}$$

□

Further, if $\frac{1}{2}\left(\eta_{i}-\eta_{j}+1\right)-{\tilde{\zeta }}_{ij}$ is greater than $\xi_{ij}$, then smaller deviation variables $d_{ij}^{+}$ are preferred. Similarly, if $\frac{1}{2}\left(\eta_{i}-\eta_{j}+1\right)-{\tilde{\zeta }}_{ij}$ less than $\xi_{ij}$, smaller deviation variables $d_{ij}^{-}$ are better, including $d_{ij}^{+},d_{ij}^{-}\geq 0$. For UPR with normal uncertain distribution, the chance constrained optimal ranking Equation (33) based on goal programming can be constructed as follows

(33) $$\begin{array}{cc} \min & \sum_{i\neq j,i,j\in N}d_{ij}^{+}+d_{ij}^{-}+\xi_{ij}\\ \;s.t.\; & \left\{\begin{array}{cc} M\left\{\frac{1}{2}\left(\eta_{i}-\eta_{j}+1\right)-{\tilde{\zeta }}_{ij}-\xi_{ij}\leq d_{ij}^{+}\right\}\geq \alpha_{ij}, & i,j\in N,\\ M\left\{-\frac{1}{2}\left(\eta_{i}-\eta_{j}+1\right)+{\tilde{\zeta }}_{ij}-\xi_{ij}\leq d_{ij}^{-}\right\}\geq \alpha_{ij}, & i,j\in N,\\ {\tilde{\zeta }}_{ij}∼N\left(e_{ij},\sigma_{ij}\right), & i,j\in N,\\ \sum_{i=1}^{n}\eta_{i}=1, & i\in N,\\ \eta_{i},d_{ij}^{+},d_{ij}^{-},\xi_{ij}\geq 0, & i,j\in N. \end{array}\right. \end{array}$$

**Theorem** **6.** *For a UPR with a normal distribution of uncertainty, the optimal ordering of Equation (33) based on chance constraints can be converted to the following equivalent form Equation (34).*

(34) $$\begin{array}{cc} \min & \sum_{i\neq j,i,j\in N}d_{ij}^{+}+d_{ij}^{-}+\xi_{ij}\\ \;s.t.\; & \left\{\begin{array}{cc} \Phi^{-1}\left(\alpha_{ij}\right)+\frac{1}{2}\left(\eta_{i}-\eta_{j}+1\right)-\xi_{ij}-d_{ij}^{+}-2e_{ij}\leq 0, & i,j\in N,\\ 2e_{ij}-\xi_{ij}-d_{ij}^{-}-\frac{1}{2}\left(\eta_{i}-\eta_{j}+1\right)-\Phi^{-1}\left(\alpha_{ij}\right)\leq 0, & i,j\in N,\\ \sum_{i=1}^{n}\eta_{i}=1, & i\in N,\\ \eta_{i},d_{ij}^{+},d_{ij}^{-},\xi_{ij}\geq 0, & i,j\in N. \end{array}\right. \end{array}$$

**Proof.**  For ${\tilde{\zeta }}_{ij}∼N\left(\mu_{ij},\sigma_{ij}\right)$, we only need to prove the following equation

(35) $$M\left\{\frac{1}{2}\left(\eta_{i}-\eta_{j}+1\right)-{\tilde{\zeta }}_{ij}-\xi_{ij}\leq d_{ij}^{+}\right\}\geq \alpha_{ij},$$

(36) $$M\left\{-\frac{1}{2}\left(\eta_{i}-\eta_{j}+1\right)+{\tilde{\zeta }}_{ij}-\xi_{ij}\leq d_{ij}^{-}\right\}\geq \alpha_{ij}.$$

These are, respectively, equivalent to

(37) $$\Phi^{-1}\left(\alpha_{ij}\right)+\frac{1}{2}\left(\eta_{i}-\eta_{j}+1\right)-\xi_{ij}-d_{ij}^{+}-2e_{ij}\leq 0,$$

(38) $$2e_{ij}-\xi_{ij}-d_{ij}^{-}-\frac{1}{2}\left(\eta_{i}-\eta_{j}+1\right)-\Phi^{-1}\left(\alpha_{ij}\right)\leq 0.$$

(39) $$\begin{array}{cc} Equation(35)\Leftrightarrow \; & M\left\{-{\tilde{\zeta }}_{ij}\leq d_{ij}^{+}-\frac{1}{2}\left(\eta_{i}-\eta_{j}+1\right)+\xi_{ij}\right\}\geq \alpha_{ij}, \end{array}$$

(40) $$\begin{array}{cc} \;\;\;\;\;\;\;\;\;\;\;\;\;\;\;\;\;\;\;\;\;\;\;\;\;\;\;\;\;\;\Leftrightarrow \; & 1-M\left\{{\tilde{\zeta }}_{ij}\leq -d_{ij}^{+}+\frac{1}{2}\left(\eta_{i}-\eta_{j}+1\right)-\xi_{ij}\right\}\geq \alpha_{ij}, \end{array}$$

(41) $$\begin{array}{cc} \;\;\;\;\;\;\;\;\;\;\;\;\;\;\;\;\;\Leftrightarrow \; & \Phi \left(\frac{1}{2}\left(\eta_{i}-\eta_{j}+1\right)-\xi_{ij}-d_{ij}^{+}\right)\leq 1-\alpha_{ij}, \end{array}$$

(42) $$\begin{array}{cc} \;\;\;\;\;\;\;\;\;\;\;\;\;\;\;\;\;\;\;\;\;\;\;\;\;\;\;\;\;\;\;\;\;\;\;\;\;\;\;\;\;\;\;\;\;\;\;\;\;\;\;\;\;\;\;\Leftrightarrow \; & {\left(1+\exp\left(\frac{\pi \left(e_{ij}-\frac{1}{2}\left(\eta_{i}-\eta_{j}+1\right)+\xi_{ij}+d_{ij}^{+}\right)}{\sqrt{3}\sigma_{ij}}\right)\right)}^{-1}\leq 1-\alpha_{ij}, \end{array}$$

(43) $$\begin{array}{cc} \;\;\;\;\;\;\;\;\;\;\;\;\;\;\;\;\;\;\;\;\;\;\;\;\;\;\;\;\;\;\;\;\;\;\;\;\;\;\;\;\;\;\Leftrightarrow \; & \exp\left(\frac{\pi \left(e_{ij}-\frac{1}{2}\left(\eta_{i}-\eta_{j}+1\right)+\xi_{ij}+d_{ij}^{+}\right)}{\sqrt{3}\sigma_{ij}}\right)\geq \frac{\alpha_{ij}}{1-\alpha_{ij}}, \end{array}$$

(44) $$\begin{array}{cc} \;\;\;\;\;\;\;\;\;\;\;\;\;\;\;\;\;\;\;\;\;\;\;\;\;\;\;\;\;\;\;\;\;\;\;\;\;\Leftrightarrow \; & e_{ij}-\frac{1}{2}\left(\eta_{i}-\eta_{j}+1\right)+\xi_{ij}+d_{ij}^{+}\geq \frac{\sqrt{3}\sigma_{ij}}{\pi }\ln\frac{\alpha_{ij}}{1-\alpha_{ij}}, \end{array}$$

(45) $$\begin{array}{cc} \;\;\;\;\;\;\Leftrightarrow \; & Equation(37).\;\;\;\;\;\;\;\;\;\;\;\;\;\;\;\;\;\;\;\;\;\;\;\;\;\;\;\;\;\;\;\;\; \end{array}$$

By the same token,

(46) $$\begin{array}{cc} Equation(36)\Leftrightarrow \; & M\left\{{\tilde{\zeta }}_{ij}\leq \frac{1}{2}\left(\eta_{i}-\eta_{j}+1\right)+\xi_{ij}+d_{ij}^{-}\right\}\geq \alpha_{ij}, \end{array}$$

(47) $$\begin{array}{cc} \;\;\;\;\;\;\;\;\;\;\;\;\;\;\;\;\;\;\;\;\;\;\;\;\;\;\;\;\;\;\;\;\;\Leftrightarrow \; & M\left\{{\tilde{\zeta }}_{ij}\leq -\frac{1}{2}\left(\eta_{i}-\eta_{j}+1\right)-\xi_{ij}-d_{ij}^{-}\right\}\leq 1-\alpha_{ij}, \end{array}$$

(48) $$\begin{array}{cc} \;\;\;\;\;\;\;\;\;\;\;\;\;\Leftrightarrow \; & \Phi \left(\frac{1}{2}\left(\eta_{i}-\eta_{j}+1\right)+\xi_{ij}+d_{ij}^{-}\right)\geq \alpha_{ij}, \end{array}$$

(49) $$\begin{array}{cc} \;\;\;\;\;\;\;\;\;\;\;\;\;\;\;\;\;\;\;\;\;\;\;\;\;\;\;\;\;\;\;\;\;\;\;\;\;\;\;\;\;\;\;\;\;\;\;\;\;\;\;\;\;\;\;\;\;\;\Leftrightarrow \; & {\left(1+\exp\left(-\frac{\pi \left(-e_{ij}+\frac{1}{2}\left(\eta_{i}-\eta_{j}+1\right)+\xi_{ij}+d_{ij}^{-}\right)}{\sqrt{3}\sigma_{ij}}\right)\right)}^{-1}\geq \alpha_{ij}, \end{array}$$

(50) $$\begin{array}{cc} \;\;\;\;\;\;\;\;\;\;\;\;\;\;\;\;\;\;\;\;\;\;\;\;\;\;\;\;\;\;\;\;\;\;\;\;\;\;\;\;\;\;\;\;\;\;\;\;\;\;\;\Leftrightarrow \; & \exp\left(-\frac{\pi \left(-e_{ij}+\frac{1}{2}\left(\eta_{i}-\eta_{j}+1\right)+\xi_{ij}+d_{ij}^{-}\right)}{\sqrt{3}\sigma_{ij}}\right)\leq \frac{1-\alpha_{ij}}{\alpha_{ij}}, \end{array}$$

(51) $$\begin{array}{cc} \;\;\;\;\;\;\;\;\;\;\;\;\;\;\;\;\;\;\;\;\;\;\;\;\;\;\;\;\;\;\;\;\;\;\;\;\;\;\;\;\Leftrightarrow \; & e_{ij}-\frac{1}{2}\left(\eta_{i}-\eta_{j}+1\right)-\xi_{ij}-d_{ij}^{-}\leq \frac{\sqrt{3}\sigma_{ij}}{\pi }\ln\frac{1-\alpha_{ij}}{\alpha_{ij}}, \end{array}$$

(52) $$\begin{array}{cc} \;\;\;\;\;\;\;\;\;\;\Leftrightarrow \; & Equation(38).\;\;\;\;\;\;\;\;\;\;\;\;\;\;\;\;\;\;\;\;\;\;\;\;\;\;\;\;\;\;\;\;\; \end{array}$$

□

## 4. Portfolio Model with Uncertain Preferences with Chance Constraints

### 4.1. Portfolio Models Considering Additive Consistency of Normal Uncertain Preferences

In Section 2 and Section 3, we review the NUPR and portfolio theory. In this section, we integrate the NUPR with the portfolio model, taking into account the theoretical foundation provided. Suppose the NUPR of an investor under the optimal portfolio outcome for *N* securities. Clearly, it is imperative for the NUPR to adhere to additive consistency in order to avoid the possibility of obtaining conflicting ranking outcomes.

Assuming that the expected return is not below $e_{0}$, and investment risk minimization, the following model ensures a uniform NUPR addition of investors.

(53) $$\begin{array}{cc} \min & \sum_{i=1}^{n}\sum_{j=1}^{n}\eta_{i}\sigma_{ij}\eta_{j}\\ \;s.t.\; & \left\{\begin{array}{cc} \sum_{i=1}^{n}\eta_{i}e_{i}\geq e_{0}, & i\in N,\\ \Phi^{-1}\left(\alpha_{ij}\right)=\frac{1}{2}\left(\eta_{i}-\eta_{j}+1\right), & i,j\in N,\\ \sum_{i=1}^{n}\eta_{i}=1, & i\in N,\\ \Phi^{-1}\left(\alpha_{ij}\right),\eta_{i}\geq 0, & i,j\in N. \end{array}\right. \end{array}$$

Compared with Equation (1), we have added the constraint $\Phi^{-1}\left(\alpha_{ij}\right)=\frac{1}{2}\left(\eta_{i}-\eta_{j}+1\right),$i,j∈N; this ensures that the minimum risk of the optimal portfolio under the weight of the NUPR meets additive consistency. In addition, the constraint itself implies that $\Phi_{ii}^{-1}\left(\alpha \right)=0.5$ and $\Phi_{ij}^{-1}\left(\alpha \right)+\Phi_{ji}^{-1}\left(1-\alpha \right)=1,i,j\in N$. Therefore, these additional constraints are not needed.

Suppose the investment risk does not exceed $\sigma_{0}^{2}$, and aim to maximize the anticipated returns. The following model ensures a uniform NUPR addition of investors.

(54) $$\begin{array}{cc} \max & \sum_{i=1}^{n}\eta_{i}e_{i}\\ \;s.t.\; & \left\{\begin{array}{cc} \sum_{i=1}^{n}\sum_{j=1}^{n}\eta_{i}\sigma_{ij}\eta_{j}\leq \sigma_{0}^{2}, & i,j\in N,\\ \Phi^{-1}\left(\alpha_{ij}\right)=\frac{1}{2}\left(\eta_{i}-\eta_{j}+1\right), & i,j\in N,\\ \sum_{i=1}^{n}\eta_{i}=1, & i\in N,\\ \Phi^{-1}\left(\alpha_{ij}\right),\eta_{i}\geq 0, & i,j\in N. \end{array}\right. \end{array}$$

Similarly to Equation (53), we add the constraint $\Phi^{-1}\left(\alpha_{ij}\right)=\frac{1}{2}\left(\eta_{i}-\eta_{j}+1\right),i,j\in N$; this ensures that when return to maximize the best portfolio under the weight of the NUPR, we meet additive consistency.

The implications of Equations (53) and (54) suggest that their approaches to addressing the portfolio problem may exhibit significant similarities, as they have only one additional constraint compared to the Equations (1) and (2), respectively. However, the significance of Equations (53) and (54) lie in their ability to achieve an NUPR that completely meets the requirement of additive consistency associated with the optimal investment plan. Consequently, investors have the opportunity to assess an NUPR in relation to their individual preferences, stabilize logical preferences, rectify illogical preferences, and establish a preference foundation and point of reference for future comparable investments.

### 4.2. A Portfolio Model Considering the Degree of Additive Consistency of Normal Uncertain Preferences

In Section 2 and Section 4, we aim to introduce the classic Markowitz portfolio model and the portfolio model that considers normal uncertain preferences with additive consistency. Both models assume that investors are fully rational, aiming to minimize the possibility of risks or maximize the potential for returns. On the other hand, in real-world scenarios of managing investment risks, investors hold a pivotal position within the system. The outcomes are considerably influenced by their perspectives, inclinations, and satisfaction towards various investment alternatives. This pertains to the application of behavioral operational theory, originating from conventional operational theory. In real-life portfolio cases, investors are often influenced by various factors such as circumstances, preferences, and experiences leading to incomplete or uncertain unique information, even contradictions at times. In the scope of the portfolio issue discussed in this paper, we expand it to a stage where investors can offer their own NUPR for various securities according to their attitudes and expertise, and combined with personal inclinations, the current investment context, and market circumstances. These NUPRs may be incomplete or inconsistent. Therefore, it is necessary to consider return rate maximization criteria along with risk minimization and consistency index maximization while developing investment portfolio models with limited knowledge of preferences.

It is assumed that the anticipated rate of return equals or is higher than $e_{0}$, and investors with a consistency index of the NUPR $Z={\left(\zeta_{ij}\right)}_{n\times n}$ are not lower than α. A model can be constructed to achieve additive consistency and minimize the risk of investment in $\mathrm{NUPR}\tilde{Z}={\left({\tilde{\zeta }}_{ij}\right)}_{n\times n}$.

(55) $$\begin{array}{cc} \min & \sum_{i=1}^{n}\sum_{j=1}^{n}\eta_{i}\sigma_{ij}\eta_{j}+\lambda \sum_{i\neq j,i,j\in N}\xi_{ij}\\ \;s.t.\; & \left\{\begin{array}{cc} \sum_{i=1}^{n}\eta_{i}e_{i}\geq e_{0}, & i\in N,\\ \Phi_{ij}^{-1}\left(\alpha \right)+\frac{1}{2}\left(\eta_{i}-\eta_{j}+1\right)-2e_{ij}-\xi_{ij}\leq 0, & i,j\in N,\\ 2e_{ij}-\xi_{ij}-\frac{1}{2}\left(\eta_{i}-\eta_{j}+1\right)-\Phi_{ij}^{-1}\left(\alpha \right)\leq 0, & i,j\in N,\\ \sum_{i=1}^{n}\eta_{i}=1, & i\in N,\\ \Phi^{-1}\left(\alpha_{ij}\right),\eta_{i},\xi_{ij}\geq 0, & i,j\in N. \end{array}\right. \end{array}$$

where λ denotes the risk aversion coefficient.

The first constraint ensures that an investor’s actual return $e_{0}$ on investment is equal to or higher than their anticipated yield. The second and third constraints from Equation (16) said $\frac{1}{2}\left(\eta_{i}-\eta_{j}+1\right)$ and $\zeta_{ij}$ deviation threshold is less than the distance between $\zeta_{ij}$. The fourth constraint ensures that weight vectors sum up to one. These constraints improve consistency in the NUPR provided by investors without unnecessarily changing their original preference information. Furthermore, these three constraints complementarily define the NUPR $Z={\left(\zeta_{ij}\right)}_{n\times n}$ (i.e., Definition 3.1). Since $\tilde{Z}={\left({\tilde{\zeta }}_{ij}\right)}_{n\times n}$ of $\Phi_{ij}^{-1}\left(\alpha \right)=2e_{ij}+\zeta_{ij}-$$\frac{1}{2}\left(\eta_{i}-\eta_{j}+1\right)$, $\Phi_{ij}^{-1}\left(\alpha \right)+\Phi_{ji}^{-1}\left(1-\alpha \right)=1,\Phi_{ii}^{-1}\left(\alpha \right)=0.5$ is directly implied. So, we do not need to be in $\tilde{Z}={\left({\tilde{\zeta }}_{ij}\right)}_{n\times n}$ to add these constraints. The fifth constraint ensures $\Phi^{-1}\left(\alpha_{ij}\right),\eta_{i}$ and $\xi_{ij}$ are negative.

Suppose that the investment risk in question does not exceed $\sigma_{0}^{2}$, and for investors with the NUPR $Z={\left(\zeta_{ij}\right)}_{n\times n}$, the consistency index is not lower than α. Construct the framework for achieving a desirable level of ensuring additive consistency and optimizing the anticipated rate of return of the NUPR $\tilde{Z}={\left({\tilde{\zeta }}_{ij}\right)}_{n\times n}$.

(56) $$\begin{array}{cc} \max & \sum_{i=1}^{n}\sum_{j=1}^{n}\eta_{i}e_{i}-\lambda \sum_{i\neq j,i,j\in N}\xi_{ij}\\ \;s.t.\; & \left\{\begin{array}{cc} \sum_{i=1}^{n}\eta_{i}\sigma_{ij}\eta_{j}\leq \sigma_{0}^{2}, & i\in N,\\ \Phi_{ij}^{-1}\left(\alpha \right)+\frac{1}{2}\left(\eta_{i}-\eta_{j}+1\right)-2e_{ij}-\xi_{ij}\leq 0, & i,j\in N,\\ 2e_{ij}-\xi_{ij}-\frac{1}{2}\left(\eta_{i}-\eta_{j}+1\right)-\Phi_{ij}^{-1}\left(\alpha \right)\leq 0, & i,j\in N,\\ \sum_{i=1}^{n}\eta_{i}=1, & i\in N,\\ \Phi^{-1}\left(\alpha_{ij}\right),\eta_{i},\xi_{ij}\geq 0, & i,j\in N. \end{array}\right. \end{array}$$

The main difference between Equations (55) and (56) is that Equation (55) prioritizes considering investment risk as the primary focus, while ensuring expected return remains within constraints. On the other hand, Equation (56) operates in the opposite manner. In terms of the remaining constraints, Equation (55) is consistent with Equation (56).

Further, if $\frac{1}{2}\left(\eta_{i}-\eta_{j}+1\right)-{\tilde{\zeta }}_{ij}$ is greater than $\xi_{ij}$, then smaller deviation variables $d_{ij}^{+}$ are preferred. Similarly, if $\frac{1}{2}\left(\eta_{i}-\eta_{j}+1\right)-{\tilde{\zeta }}_{ij}$ is less than factor $\xi_{ij}$, then smaller deviation variables $d_{ij}^{-}$ are preferred, including when $d_{ij}^{+},d_{ij}^{-}\geq 0$. For UPR with normal uncertain distributions, a portfolio model can be constructed using chance constraints based on goal programming as follows. Assuming ensuring that the anticipated return rate does not fall below $e_{0}$, the following model can be used to obtain an NUPR $\tilde{Z}={\left({\tilde{\zeta }}_{ij}\right)}_{n\times n}$, which meets the requirement of additive consistency while optimizing the overall coherence degree of the NUPR $Z={\left(\zeta_{ij}\right)}_{n\times n}$ given by investors.

(57) $$\begin{array}{cc} \min & \sum_{i=1}^{n}\sum_{j=1}^{n}\eta_{i}\sigma_{ij}\eta_{j}+\lambda \sum_{i\neq j,i,j\in N}\left(d_{ij}^{+}+d_{ij}^{-}+\xi_{ij}\right)\\ \;s.t.\; & \left\{\begin{array}{cc} \sum_{i=1}^{n}\eta_{i}e_{i}\geq e_{0}, & i\in N,\\ \Phi^{-1}\left(\alpha_{ij}\right)+\frac{1}{2}\left(\eta_{i}-\eta_{j}+1\right)-\xi_{ij}-d_{ij}^{+}-2e_{ij}\leq 0, & i,j\in N,\\ 2e_{ij}-\xi_{ij}-d_{ij}^{-}-\frac{1}{2}\left(\eta_{i}-\eta_{j}+1\right)-\Phi^{-1}\left(\alpha_{ij}\right)\leq 0, & i,j\in N,\\ \sum_{i=1}^{n}\eta_{i}=1, & i\in N,\\ \Phi^{-1}\left(\alpha_{ij}\right),\eta_{i},d_{ij}^{+},d_{ij}^{-},\xi_{ij}\geq 0, & i,j\in N. \end{array}\right. \end{array}$$

The fundamental difference between Equations (55) and (57) lies in the incorporation of additional segmentation variables, which results in a deviation from the expected return of no less than $e_{0}$ and a minimum risk deviation. However, in terms of other constraints, Equation (57) remains consistent with Equation (55).

Assuming that the investment risk does not exceed $\sigma_{0}^{2}$, we can construct the following model to derive an NUPR $\tilde{Z}={\left({\tilde{\zeta }}_{ij}\right)}_{n\times n}$, aiming to meet the requirements of investors. We seek a solution that achieves both additive consistency and optimizes the consistency level of the NUPR $Z={\left(\zeta_{ij}\right)}_{n\times n}$.

(58) $$\begin{array}{cc} \max & \sum_{i=1}^{n}\sum_{j=1}^{n}\eta_{i}e_{i}-\lambda \sum_{i\neq j,i,j\in N}\left(d_{ij}^{+}+d_{ij}^{-}+\xi_{ij}\right)\\ \;s.t.\; & \left\{\begin{array}{cc} \sum_{i=1}^{n}\eta_{i}\sigma_{ij}\eta_{j}\leq \sigma_{0}^{2}, & i\in N,\\ \Phi^{-1}\left(\alpha_{ij}\right)+\frac{1}{2}\left(\eta_{i}-\eta_{j}+1\right)-\xi_{ij}-d_{ij}^{+}-2e_{ij}\leq 0, & i,j\in N,\\ 2e_{ij}-\xi_{ij}-d_{ij}^{-}-\frac{1}{2}\left(\eta_{i}-\eta_{j}+1\right)-\Phi^{-1}\left(\alpha_{ij}\right)\leq 0, & i,j\in N,\\ \sum_{i=1}^{n}\eta_{i}=1, & i\in N,\\ \Phi^{-1}\left(\alpha_{ij}\right),\eta_{i},d_{ij}^{+},d_{ij}^{-},\xi_{ij}\geq 0, & i,j\in N. \end{array}\right. \end{array}$$

The main difference between Equations (56) and (58) lies in the inclusion of additional segmentation variables, which enables the utilization of positive deviations exceeding $\sigma_{0}^{2}$ while minimizing negative deviations. In terms of other constraints, Equation (58) remains consistent with Equation (56).

### 4.3. Portfolio Model Comparison and Selection Process Analysis

In the Section 2, we review the classical Markowitz portfolio model. Then, the portfolio model with uncertain preferences with chance constraints is presented in the Section 4. These models have different features, strengths, and weaknesses and are adapted to different environments.

The primary distinction among the eight models lies in their objective and characteristic constraints. Equations (1), (53), (55), and (57) aim to minimize risk, while Equations (2), (54), (56) and (58) aim to maximize return.

In terms of pros and cons, Equations (1) and (2) do not take into account the preference relationships of investors towards different investment objects. Hence, these portfolio selection models can be applied without imposing any preference constraints. However, if an investor requires consistency in the preferences they have while making investment decisions and prefers a certain level of information, Equations (53) and (54) offer an NUPR *Z* that ensures additive consistency. Additionally, when considering the degree of preference information consistency, Equations (55)–(58) optimize single-deviation variable risk and return as well as three-deviation variable risk and return, respectively. We obtain $\frac{1}{2}\left(\eta_{i}-\eta_{j}+1\right)$ under additive consistency with deviation threshold $\zeta_{ij}$, less than the distance between $\zeta_{ij}$ of the NUPR *Z* representing the weight vector on behalf of investment proportion. The model constructed in this paper primarily considers the preference information relationship within portfolios by linking NUPRs to portfolio models through weight vectors. This provides a novel method for studying behavioral investments.

**Figure 3:**
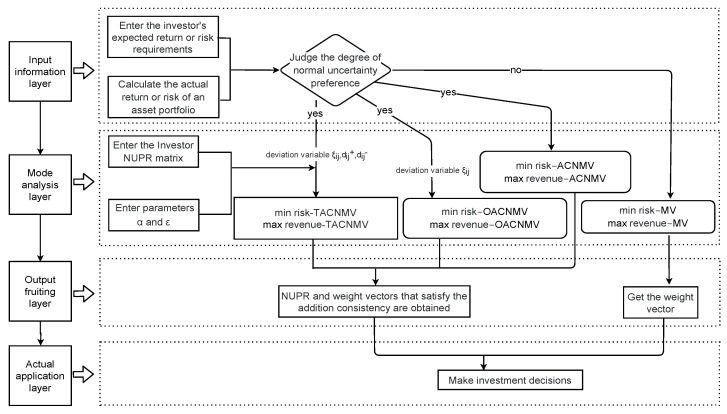
Portfolio model selection process.

The investment process is illustrated in Figure 3, which depicts various conditions and scenarios. It encompasses four key stages. To make investment decisions, it is necessary to have access to important information such as $e_{0},e_{i},\sigma_{0}$ and $\sigma_{ij}$ (please note that this information may vary based on the circumstances and specific model employed). If there are no specific investment preferences, classical Equations (1) and (2) can be solved directly. The four layers involved in this process include the layer for input and evaluation, the layer for desired outcome, the layer for model representation, and the layer for result generation. When the investor seeks to ensure consistency in their investment preferences and prioritizes information accuracy, Equations (53) and (54) yield an NUPR *Z* that satisfies additive consistency. In addition, when the degree of preference information consistency needs to be considered, Equations (55)–(58) are optimized for single-deviation variable risk and return and three-deviation variable risk and return, respectively. We obtain an NUPR *Z* for which the distance between $\frac{1}{2}\left(\eta_{i}-\eta_{j}+1\right)$ and $\zeta_{ij}$ does not exceed the deviation threshold $\zeta_{ij}$ under additive consistency, and the weight vector representing the proportion of investment at this time. Finally, the investment strategy is implemented based on the weight vector.

## 5. Empirical Analysis

In this section, the effectiveness and efficiency of the proposed model were tested by selecting different numbers of stocks from various industries included in the SSE 50 Index.

### 5.1. Data Preprocessing

In this section, the core objective of the data preprocessing stage is to screen and optimize stock samples according to the goals of the investment portfolio, ensuring that the selected stocks can effectively verify the applicability and reliability of the model. During the preliminary analysis phase (as shown in Section 5.2 and Section 5.3), we selected four stocks for the experiment. 600028 represents the refining and chemical industry, 600089 represents power transmission and transformation equipment, 601398 represents large state-owned banks, and 601628 represents insurance. The main reasons for this selection are to simplify the model and enhance the feasibility of the experiment. Firstly, a smaller-scale stock portfolio facilitates in-depth analysis. Especially in the initial research stage, the selection of four stocks makes the model’s calculation and analysis more concise and clear. Secondly, this selection takes into account industry representativeness, ensuring that it covers the characteristics of different industries. This enables the research to compare the performance of stocks across industries and further verify the risk and return relationships of the model among different securities.

After completing the preliminary experiments on four stocks, in order to further verify the effectiveness of the model and enhance the universality of the experimental results, we decided to expand the sample to 16 stocks (as shown in Section 5.4). This decision was based on two main factors. Firstly, expanding the sample size can provide richer experimental data, which helps to improve the robustness of the model results. Secondly, the increased stock samples can cover a wider range of industries and market backgrounds, thereby enhancing the exploration of the risk-return relationship of different securities. During the data preprocessing process, we first screened the constituent stocks of the SSE 50 Index. Since some companies had situations such as mergers, suspensions of trading, or delistings, which made it impossible to obtain complete historical data, we excluded these stocks. Then, considering the goal of maximizing the return of the investment portfolio, we removed the stocks with negative expected returns. Finally, 20 stocks remained. To further optimize the industry representativeness and risk-return characteristics of the sample, we selected a representative single stock from each industry to ensure that stocks with higher returns were chosen at the same risk level. Eventually, 16 stocks that met the research objectives were obtained, as shown in Table 1.

### 5.2. Background Description and Parametric Assumptions

This section presupposes that an individual is ready to allocate funds into a portfolio consisting of four securities. To facilitate the process, we have opted for four stocks from diverse sectors within the SSE 50 index as potential investment options. The securities codes are Sinopec (600028), TBEA (600089), ICBC (601398), and China Life Insurance (601628). Labeled $\zeta_{1},\;\zeta_{2},\;\zeta_{3},\;\zeta_{4}$ (i.e., N=4), the weekly closing prices of these four securities from April 2021 to March 2024 are obtained, and their expected returns are calculated.

We calculated the expected rate of return of securities for four e=(0.0023,0.0046,0.0021,0.0015), as well as the matrix $D\left(R\right)={\left(\sigma_{ij}\right)}_{n\times n}\left(1\times 10^{-2}\right)$ that represents the relationship between them.

$${\left(\sigma_{ij}\right)}_{4\times 4}=\left(\begin{array}{cccc} 0.2000 & 0.0163 & 0.0016 & 0.0080\\ 0.0163 & 0.3390 & 0.0003 & 0.0017\\ 0.0016 & 0.0003 & 0.1207 & 0.0028\\ 0.0080 & 0.0017 & 0.0028 & 0.2969 \end{array}\right)$$

In behavioral finance, investor preferences are influenced not only by rational economics, such as Markowitz’s mean-variance theory, but also by psychological factors and behavioral biases. By considering factors in behavioral finance like investor risk aversion, loss aversion, and expected return, we can construct a matrix of potential uncertain preference relations for investors. For instance, when faced with uncertainty, investors tend to rely on familiar stocks within their own industry or those they have previously purchased.

### 5.3. Comparison of Results and Sensitivity Analysis—Using the Risk Minimization Model as an Example

The paper proposes the ACNMV model as shown in Equation (53), the OACNMV model as given in Equation (55), and the TACNMV model as presented in Equation (57) for the classical MV portfolio model, aiming to minimize investment risk while ensuring a constant return rate or a minimum threshold. However, the OACNMV model incorporates chance constraints for the NUPR *Z* that satisfy additive consistency, whereas the TACNMV model introduces subdivision bias variables as an enhancement over the OACNMV model. A comparative analysis of these three models is conducted. With $e_{0}=0.002$ and λ=1 set, Table 2 presents the results obtained from these models.

The weight vectors adopted in the example (Section 5.2) are derived from the optimization frameworks of the MV, ACNMV, OACNMV, and TACNMV models, each minimizing portfolio risk under distinct constraints. For the MV model, weights are determined by solving a quadratic program with expected return and budget constraints. The ACNMV model extends this by enforcing additive consistency on the NUPR matrix *Z*, while the OACNMV and TACNMV models further incorporate chance constraints and subdivision bias variables, respectively. The consistency of these weights is validated by: (a) ensuring numerical stability via symmetric covariance matrices (e.g., $\Sigma \leftarrow 0.5\left(\Sigma +\Sigma^{⊤}\right)$); (b) verifying feasibility of the target return $e_{0}=0.002$ within the range of asset returns; and (c) confirming non-negativity and unit-sum constraints. The results in Table 2 reflect these rigorous computations, with risk values ($\sum_{i,j}\eta_{i}\sigma_{ij}\eta_{j}$) demonstrating the trade-offs between model complexity and risk minimization.

Table 2 demonstrates significant disparities in asset weight allocation and portfolio risk across various portfolio optimization strategies. Firstly, the minimum variance portfolio (MV) represents a classical approach aimed at minimizing overall portfolio risk by appropriately distributing asset weights. In this strategy, the weight of asset $\eta_{2}$ is zero, indicating that without any additional constraints, $\eta_{2}$ does not significantly contribute to the optimal combination of risks or may be excluded due to its risk characteristics. However, with the implementation of constrained optimization portfolios (such as ACNMV, OACNMV, TACNMV), the weight of $\eta_{2}$ notably increases to 0.1456. This adjustment can be interpreted as a result of introducing specific constraints such as tail risk management, liquidity requirements, or individual asset limits into the portfolio optimization model, which prompts a reassessment of $\eta_{2}$’s importance. The weights assigned to other assets like $\eta_{1},\eta_{3}$, and $\eta_{4}$ are also slightly modified in order to meet new optimization conditions and achieve an enhanced balance between risk and return.

**Figure 4:**
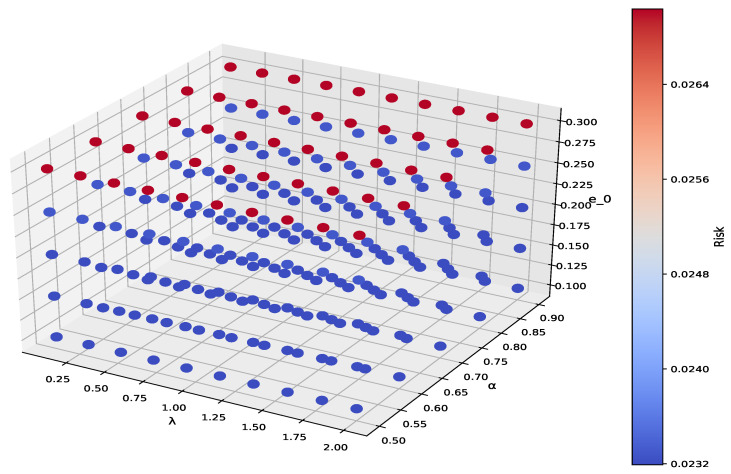
Values of minimal risk for various α and λ parameters.

The introduction of the constraint reduces the overall portfolio risk from 0.0006 to 0.0005, and although this change may seem marginal, it has a positive impact on long-term cumulative returns in large portfolios due to its magnitude of risk reduction. This phenomenon highlights the essential role that optimization constraints play in risk management. By integrating more advanced optimization methods, our enhanced strategy provides better risk control than traditional MV portfolios while improving adaptability and stability across various market conditions. Figure 4 demonstrates a sensitivity analysis of our proposed model.

The findings are applicable to $e_{0}=0.002$, α=0.05, and λ=1. The models ACNMV, OACNMV, and TACNMV represent the primary models proposed in this paper and incorporate all three parameters. Consequently, they are now subjected to a sensitivity analysis. In relation to this matter, Figure 4 demonstrates the minimum risk values across various α and λ.

The sensitivity analysis of this code demonstrates the volatility of portfolio risk under different parameter conditions. In the study, key parameters like risk aversion coefficient λ, reliability level α, and target return threshold $e_{0}$ were adjusted to analyze their effects on portfolio risk. Results show that lower risk aversion and higher return targets increase volatility, while higher risk aversion and lower reliability reduce risk. Investors should adjust these parameters based on their risk tolerance and return goals to optimize their strategy. Balancing risks and benefits improves decision making.

### 5.4. Out-of-Sample Validation and Model Expansion

This section assumes that investors are ready to allocate funds to an investment portfolio consisting of 16 securities. As shown in the method of selecting out-of-sample data in Section 5.1, we selected 16 stocks from different sectors of the SSE 50 Index as potential investment options. The security codes are 600028, 600050, 600089, 600111, 600150, 600406, 600690, 600809, 600900, 601225, 601398, 601628, 601668, 601669, 601899, and 601919. These 16 securities are labeled as $\zeta_{1}$, $\zeta_{2}$, …, $\zeta_{16}$ (i.e., N=16). We obtained their weekly closing prices from April 2021 to March 2024 and calculated their expected returns.

To verify the out-of-sample data stability of the ACNMV model, OACNMV model, and TACNMV model proposed in this paper, the experimental parameter settings are kept consistent with those in Section 5.2. The aim is to minimize the investment risk while ensuring a constant rate of return or reaching the minimum threshold. Table 3 presents the results obtained from these models, and a comparative analysis of these three models is carried out.

**Figure 5:**
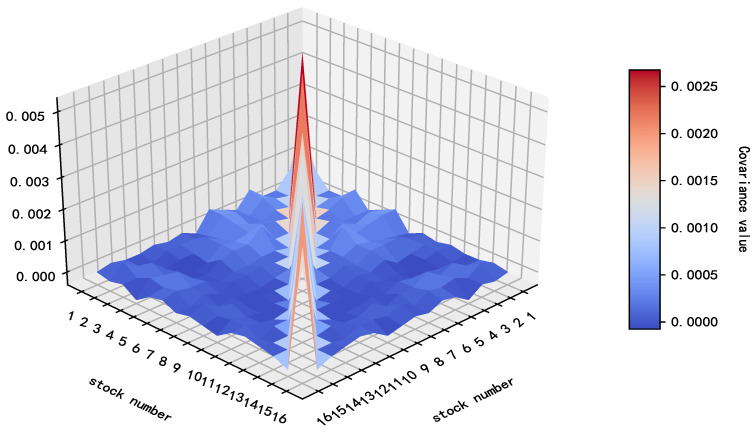
The relationship matrix D(R) among 16 kinds of securities.

The expected returns of 16 securities are e=(0.0023,0.0016,0.0046,0.0058,0.0048,0.0055,0.0048,0.0068,0.0028,0.0058,0.0021,0.0015,0.0016,0.0023,0.0029,0.0027), and the matrix representing the relationships among them (as shown in Figure 5) is $D\left(R\right)={\left(\sigma_{ij}\right)}_{n\times n}$.

Based on the out-of-sample analysis in Table 3, this study examines asset allocation and risk performance under various portfolio optimization strategies. The results show that adding constraints alters the asset weight distribution and improves the portfolio risk structure. Specifically, in the traditional mean-variance portfolio (MV), the weight of asset $\eta_{2}$ is zero, indicating its low contribution to risk minimization without constraints. However, in constrained strategies (ACNMV, OACNMV, TACNMV), $\eta_{2}$’s weight increases to 0.0285–0.0286, highlighting the significant impact of constraints on asset selection. These strategies incorporate non-traditional factors such as tail risk and liquidity, allowing assets excluded by MV to gain meaningful weights.

Further analysis shows that the constrained optimization strategy adjusts asset weights differently. High-risk asset $\eta_{9}$, already weighted at 0.1483 in the MV strategy, increases to 0.1891 in the constrained strategy, a 27.5% rise. Conversely, asset $\eta_{5}$ decreases from 0.0198 in the MV strategy to zero in the constrained strategy. This asymmetry confirms that constrained optimization involves more than simple weight redistribution. Notably, all constrained strategies (ACNMV, OACNMV, TACNMV) yield nearly identical weight configurations (with differences under 0.0001), demonstrating stability in asset selection under various constraints.

From the perspective of risk indicators, the constrained strategy offers significant advantages. The total portfolio risk decreases from 0.000263 under mean-variance (MV) to 0.000191–0.000192 under the constrained strategy, a relative reduction of 27.2%. In financial practice, this is equivalent to an annualized risk reduction of approximately ¥272,000 for a ¥1 billion portfolio. Risk reduction occurs primarily through two mechanisms: excluding extreme risk assets and optimizing the covariance structure among assets. Out-of-sample tests confirm that the constrained optimization strategy reduces risk levels while maintaining portfolio stability, providing empirical support for institutional investors in complex market environments.

## 6. Conclusions

The study promotes portfolio decision making under uncertainty by proposing a new priority model for the uncertain preference relation (UPR) with chance constraints and an additive consistency model. It extends the Markowitz theory to handle situations where investors’ preferences are incomplete or inconsistent. Theoretically, we integrate uncertainty theory with portfolio optimization and introduce chance constraints based on UPR to quantify epistemic uncertainty. Methodologically, our additive consistency model ensures reasonable preference aggregation in complex financial environments. Empirical verification using Shanghai Stock Exchange 50 stocks confirms the dual effectiveness of this model in maximizing returns and minimizing risks. Sensitivity analysis highlights its adaptability to dynamic market conditions, providing a reliable alternative to traditional stochastic methods. One limitation of this study lies in its reliance on the basic normal uncertain preference relation (NUPR), which motivates future research on quasi-normal UPR for actual market distributions and diverse preference information (such as fuzzy or heterogeneous information) to optimize decision making. These research findings collectively bridge the gap between uncertain preference theory and actual portfolio selection, adapting to the increasingly volatile financial landscape.

## Figures and Tables

**Table 1:** Stock information and expected weekly returns.

Stock Code	Expected Weekly Rate of Return	Industry Affiliation
600028	0.0023	Refining and petrochemical industry
600050	0.0016	Communication services
600089	0.0046	Power transmission and transformation equipment
600111	0.0058	Rare earths
600150	0.0048	Navigation equipment III
600406	0.0055	Power grid automation equipment
600690	0.0048	White goods
600809	0.0068	Chinese liquor III
600900	0.0028	Electric power
601225	0.0058	Steam coal
601398	0.0021	Large state-owned banks III
601628	0.0015	Insurance III
601668	0.0016	House construction II
601669	0.0023	Infrastructure construction
601899	0.0029	Copper
601919	0.0027	Shipping

**Table 2:** Optimal investment weights for the models MV, ACNMV, OACNMV, and TACNMV.

Weight/Risk	MV	ACNMV	OACNMV	TACNMV
$\eta_{1}$	0.2583	0.2468	0.2468	0.2468
$\eta_{2}$	0.0000	0.1456	0.1456	0.1456
$\eta_{3}$	0.4889	0.4381	0.4381	0.4381
$\eta_{4}$	0.2528	0.1696	0.1696	0.1696
$\sum_{i=1}^{n}\sum_{j=1}^{n}\eta_{i}\sigma_{ij}\eta_{j}$	0.0006	0.0005	0.0005	0.0005

**Table 3:** Out-of-sample validation results.

Weight/Risk	MV	ACNMV	OACNMV	TACNMV
$\eta_{1}$	0.0129	0.0640	0.0640	0.0640
$\eta_{2}$	0.0000	0.0286	0.0286	0.0285
$\eta_{3}$	0.0545	0.0364	0.0364	0.0363
$\eta_{4}$	0.0484	0.0218	0.0218	0.0218
$\eta_{5}$	0.0198	0.0000	0.0000	0.0000
$\eta_{6}$	0.0891	0.0393	0.0393	0.0392
$\eta_{7}$	0.0786	0.0357	0.0357	0.0357
$\eta_{8}$	0.0982	0.0305	0.0305	0.0304
$\eta_{9}$	0.1483	0.1891	0.1891	0.1891
$\eta_{10}$	0.1225	0.0663	0.0663	0.0662
$\eta_{11}$	0.1204	0.1631	0.1631	0.1631
$\eta_{12}$	0.0474	0.0871	0.0870	0.0870
$\eta_{13}$	0.0658	0.1215	0.1215	0.1215
$\eta_{14}$	0.0186	0.0344	0.0344	0.0343
$\eta_{15}$	0.0369	0.0447	0.0447	0.0447
$\eta_{16}$	0.0386	0.0375	0.0376	0.0382
$\sum_{i=1}^{n}\sum_{j=1}^{n}\eta_{i}\sigma_{ij}\eta_{j}$	0.000263	0.000192	0.000192	0.000191

## Data Availability

The original data presented in the study are openly available in Choice Data at https://choice.eastmoney.com/.
